# Analgesic Effect of Photobiomodulation on *Bothrops Moojeni* Venom-Induced Hyperalgesia: A Mechanism Dependent on Neuronal Inhibition, Cytokines and Kinin Receptors Modulation

**DOI:** 10.1371/journal.pntd.0004998

**Published:** 2016-10-17

**Authors:** Nikele Nadur-Andrade, Camila Squarzoni Dale, Victoria Regina da Silva Oliveira, Elaine Flamia Toniolo, Regiane dos Santos Feliciano, José Antonio da Silva Jr., Stella Regina Zamuner

**Affiliations:** 1 Universidade Nove de Julho, São Paulo, São Paulo, Brazil; 2 Department of Anatomy, Institute of Biomedical Sciences, University of São Paulo, São Paulo, São Paulo, Brazil; Universidad de Costa Rica, COSTA RICA

## Abstract

**Background:**

Envenoming induced by *Bothrops* snakebites is characterized by drastic local tissue damage that involves an intense inflammatory reaction and local hyperalgesia which are not neutralized by conventional antivenom treatment. Herein, the effectiveness of photobiomodulation to reduce inflammatory hyperalgesia induced by *Bothrops moojeni* venom (Bmv), as well as the mechanisms involved was investigated.

**Methodology/Principal Findings:**

Bmv (1 μg) was injected through the intraplantar route in the right hind paw of mice. Mechanical hyperalgesia and allodynia were evaluated by von Frey filaments at different time points after venom injection. Low level laser therapy (LLLT) was applied at the site of Bmv injection at wavelength of red 685 nm with energy density of 2.2 J/cm^2^ at 30 min and 3 h after venom inoculation. Neuronal activation in the dorsal horn spinal cord was determined by immunohistochemistry of Fos protein and the mRNA expression of IL-6, TNF-α, IL-10, B1 and B2 kinin receptors were evaluated by Real time-PCR 6 h after venom injection. Photobiomodulation reversed Bmv-induced mechanical hyperalgesia and allodynia and decreased Fos expression, induced by Bmv as well as the mRNA levels of IL-6, TNF-α and B1 and B2 kinin receptors. Finally, an increase on IL-10, was observed following LLLT.

**Conclusion/Significance:**

These data demonstrate that LLLT interferes with mechanisms involved in nociception and hyperalgesia and modulates Bmv-induced nociceptive signal. The use of photobiomodulation in reducing local pain induced by Bothropic venoms should be considered as a novel therapeutic tool for the treatment of local symptoms induced after bothropic snakebites.

## Author Summary

Envenoming caused by Bothrops snakes is characterized by drastic local tissue damage involving hemorrhage, blistering, myonecrosis, prominent inflammatory response and intense pain. The most effective treatment for Bothrops snakebites is antivenom therapy, which is very efficient in reversing systemic effects of envenomation but not the severe local effects. Thus, there exists a need to find novel complementary therapies that may further assist in the prevention or even counteract the severe local effects of bothrops snakebite. Several studies have shown the effectiveness of photobiomodulation in reducing local effects induced by Bothropic venoms, however its mechanisms still remain unknown. In this study, we analyzed the effectiveness of photobiomodulation in reducing BmV-induced mechanical allodynia and hyperalgesia as well as part of the mechanisms involved in such effect. Results demonstrate that photobiomodulation reduces venom-induced mechanical allodynia and hyperalgesia and this effect depends on a decrease of nociceptor activation at the spinal cord level and by a modulation of pro- and anti- inflammatory cytokines as well as kinin receptors at mRNA transcriptional levels. These findings make phtobiomodulation a promising candidate to be associated to antivenom therapy for the treatment of the local response induced by Bothrops venoms.

## Introduction

Bothropic envenomation is characterized by severe local manifestation associated with oedema, myonecrosis, hemorrhage and intense pain [1–4] caused by the toxic action of venom components and aggravated by induced-inflammation. The local effects induced by bothropic venoms are the result of multifactorial and synergistic actions of toxins, which are still poorly understood. *Bothrops moojeni* is a venomous snake responsible for most of the snakebites in the Central region of Brazil [5]. Despite the medical importance, there are only a few studies related to the local inflammatory reaction caused by *Bothrops moojeni* venom (Bmv). In this sense, the literature shows that in the accidents caused by these snakes serious local complications occur, including a prominent edema formation, intense pain, swelling and pallor, which may develop into more severe outcomes such as muscle mass loss, neuropathy, and amputation [6, 7].

Currently, the most effective treatment for Bothrops snakebites accidents is the antivenom therapy (AV). However, although AV has proven to be effective in reversal the systemic response, its administration does not prevent local effects and resultant disabilities [3]. Consequently, there is a need to find therapeutic approaches associated with AV treatment that can be effective in reducing the local effects caused by Bothrops snakes envenoming in order to minimize or prevent the progression to a severe clinical status observed after Bothrops snakebites [8, 9].

Photobiomodulation is a form of light that triggers biochemical changes within cells, where the photons are absorbed by cellular photoreceptors and triggers chemical alterations [10]. The mechanisms of photobiomodulation essentially rely on particular visible red and infrared light waves in photoreceptors within sub-cellular components, particularly the respiratory chain within mitochondrial membranes due to the activation of various transcription factors by the immediate chemical signaling molecules produced from mitochondrial stimulation [11]. The most important of these signaling molecules are thought to be Adenosine Triphosphate (ATP), cyclic-AMP, nitric oxide (NO) and Reactive Oxygen Species (ROS) [12].

Many studies have demonstrated analgesic and anti-inflammatory effects provided by photobiomodulation in both experimental [13, 14] and clinical trials [15, 16]. Photobiomodulation has also proven to be an interesting and efficient complementary alternative for the treatment of local effects caused by bothropic venom through the ability of decreasing the observed local effects, such as myonecrosis [17, 18]; inflammation [19–22] hemorrhage [21] and pain [20, 23]. In this context, we have recently demonstrated that photobiostimulation with LLLT and light emitting diode (LED) reverse edema formation, local hemorrhage and inflammatory hyperalgesia induced by *Bohtrops moojeni* venom (BmV) in mice [18, 24].

Although some studies have demonstrated the effectiveness of photobiomodulation in reducing hyperalgesia and allodynia induced by bothropic venom, the mechanism involved in this effect still remains unknown. In this context, the present experiments were designed to investigate the antinociceptive effect of photobiomodulation on BmV-induced allodynia and hyperalgesia and to explore possible underlying mechanisms.

## Materials and Methods

### Animals

Male Swiss mice weighing 20–25 g, age-matched, were used throughout this study. Animals were maintained under controlled light cycle (12/12 h) and temperature (21 ± 2°C) with free access to food and water.

### Ethics Statement

All animal experimentation protocols received the approval by the Ethics Committee on the Use of Animals at of Hospital Sírio-Libanês (Protocol no. (CEUA 2010/01), in agreement with Brazilian federal law (11.794/2008, Decreto n° 6.899/2009). We followed institutional guidelines on animal manipulation, adhering to the “Principles of Laboratory Animal Care” (National Society for Medical Research, USA) and the “Guide for the Care and Use of Laboratory Animals” (National Academy of Sciences, USA).

### Venom and Antivenom

*Bothrops moojeni* venom (Bmv) was supplied by the Serpentarium of the Center of Studies of Nature at UNIVAP. Bmv was lyophilized, kept refrigerated at 4°C and diluted in sterile saline solution (0.9%) immediately before use. Bmv was injected into the subplantar surface of the right hind paw at the concentration of 1.0 μg/50 μL. Equine antivenom (AV) used in the experiments was a polyvalent Bothrops AV (lot# 990504–18) raised against a pool of venom from *B*. *alternatus*, *B*. *jararaca*, *B*. *jararacussu*, *B*. *cotiara*, *B*. *moojeni* and *B*. *neuwiedi* obtained from the Butantan Institute (São Paulo, SP, Brazil). AV was injected through the intravenous route (0.2 μL of AV diluted in saline; final volume of 50 μL, considering that 1 mL of AV neutralizes 5 mg of Bothropic venom [25] 30 min after BmV injection.

### Mechanical Hyperalgesia and Tactile Allodynia

Hyperalgesia and allodynia of the hind paw were assessed as described by Takasaki et al. [17]. Mice were placed individually in plastic cages with a wire bottom, which allowed access to their paws. To reduce stress, mice were habituated to the experimental environment one day before the first measurement. At the day of the test, the animals were placed in the cages 30 min before the beginning of each measurement and received an injection of 1.0 μg of crude Bmv diluted in 50 μL of sterile saline into the subplantar surface of the right hind paw. Control group animals received the same volume of sterile saline. Von Frey filaments with bending forces of 0.407 g (3.61 filament—allodynia stimulus), 0.692 g and 1.202 g (3.84 and 4.08 filaments—hyperalgesia stimulus) were pressed perpendicularly against the plantar skin and held for 5 s, at 1, 3, 6 and 24 h after venom injection. A stimulation of the same intensity was applied three times to each hind paw at intervals of 5 s. The responses to these stimuli were ranked as follows: 0, no response; 1, move away from von Frey filament and 2, immediate flinching or licking of the hind foot. The nociceptive score was calculated as follows:

$$\text{Nociceptive score }\left(\%\right)\;=\;\frac{\Sigma \left(\text{average score of each animal}\right)\;x\;100}{2\;x\text{ number tested animals}}$$

Animals were returned to their home cages with free access to food and water between the 1 and 3 h, 3 and 6 h and 6 and 24 h measurements.

### Light Source, Dose and Treatment

A low-level semiconductor Ga-As laser, Theralaser D.M.C. (São Carlos, SP, Brazil), operating with a wavelength of red 685 nm, was used through the experiments with a beam spot of 0,2 cm^2^ and an output power of 30 mW, energy density of 2.2 J/cm^2^ and exposure time of 15 s. Laser doses, low enough to avoid any thermal effect, were chosen on the basis of previous study from our laboratory [18]. Animals were gently manually restrained and the LLLT was applied to the same area as the injection of Bmv or saline solution. A control group was treated using the same experimental procedure but with the laser turned off. Animals were irradiated 30 min and 3 h after subplantar injection of either Bmv or saline and were immediately returned to their home cages with free access to food and water after each application.

Experiments were conducted in an environment with partial obscurity to not suffer interference from external light. The output power of the laser equipment was measured using the Laser Check1power meter (MM Optics, São Carlos, Brazil).

### Immunohistochemistry

Six hours after the intraplantar (i.pl.) injection of Bmv or saline, mice were deeply anesthetized with ketamine hydrochloride (100 mg/kg) and xylazine (10 mg/kg) and transcardially perfused with phosphate-buffered saline and 4% paraformaldehyde in 0.1 M phosphate buffer (PB; pH 7.4). The spinal cord (L4 and L5) was removed, left in the same fixative for 5–8 h and then cryoprotected overnight in 30% sucrose. Thirty μm frozen sections were immunostained for Fos expression. The spinal cord sections were incubated free floating with a rabbit polyclonal antibody against the nuclear protein which is the product of the early response gene c-fos (Ab-5; Calbiochem, CA/USA), and diluted 1:1000 in PB containing 0.3% Triton X-100 plus 5% of normal goat serum. Incubation with the primary antibody was conducted overnight at 24°C. After three washes (10 min each) in PB, the sections were incubated with biotinylated goat anti-rabbit sera (Vector Labs, Burlingame, CA) diluted 1:200 in PB for 2 h at 24°C. The sections were washed again in PB and incubated with the avidin-biotin-peroxidase complex (ABC Elite; Vector Labs). After the reaction with 0.05% 3–3’ diaminobenzidine and a 0.01% solution of hydrogen peroxide in PB and intensification with 0.05% osmium tetroxide in water, the sections were mounted on gelatin- and chromoalumen-coated slides, dehydrated, cleared, and coversliped. The material was then analyzed on a light microscope, and digital images were collected. A quantitative analysis of the immunolabeled material was analyzed using a light microscope and the NIS Elements F3.0 Image analysis system (Nikon Instruments Inc., USA). A quantitative analysis was performed on the density of nuclei representative of thle immunoreactivity for Fos (Fos-IR) in: a) the dorsal horn of the spinal cord (DHSC; superficial laminae-I to IV according to the classification of Rexed. Measurements were taken from 10 different sections for each animal analyzed, including areas that were defined for each structure by using a 20 x objective for the DHSC. Measurements were performed with the program Image J and the operator was blinded to the animal treatment group.

### Real-Time Quantitative Polymerase Chain Reaction (PCR)

Total RNA was isolated from subplantar muscles and spinal cord by TRIzol reagent (Gibco BRL, Gaithersburg, MD), according to the manufacturer's protocol. RNA was subjected to DNase I digestion, followed by reverse transcription to cDNA, as previously described [26]. PCR was performed in a 7000 Sequence Detection System (ABI Prism, Applied Biosystems, Foster City, CA) using the SYBRGreen core reaction kit (Applied Biosystems). Primers used are described in Table 1.

**Table 1:** PCR primer sequences.

Name	Sequence (5'-3')
**Interlekin-6**	5'-GAGGAGACTTCACAGAGGAT-3'
**Interleukin -10**	5’-TTGAACCACCCGGCATCTAC-3’
**TNF-α**	5′-AAATGGGCTCCCTCTATCAGTTC-3′
**GAPDH**	5’-TGCACCACCAACTGCTTAGC-3’
**kinin B1**	5′-CCAGGGTTCGTCATCACTATCTG-3′
**kinin B2**	5′-CCCTTCCTCTGGGTCCTCTT- 3′

Quantitative values for IL-6, IL-10, TNF-α, kinin B1 and B2 receptors, CAPDH and mRNA transcription were obtained from the threshold cycle number, where the increase in the signal associated with an exponential growth of PCR products begins to be detected. Melting curves were generated at the end of every run to ensure product uniformity. The relative target gene expression level was normalized on the basis of GADPH expression as endogenous RNA control [27]. Results are expressed as a ratio relative to the sum of GAPDH transcript levels as internal control.

### Statistical Analysis

Results were expressed as the mean±SEM. Statistical analyses of data were generated by using GraphPad Prism, version 4.02 (GraphPad). A value of p<0.05 indicated a significant difference. Statistical comparison of more than two groups was performed using analysis of variance (ANOVA), followed by Bonferroni’s test. Statistical comparison for treatment over time was performed using two way ANOVA followed by Bonferroni’s test.

## Results

### Effect of Photobiomodulation on Bmv-Induced Mechanical Allodynia and Hyperalgesia

**Fig 1:**
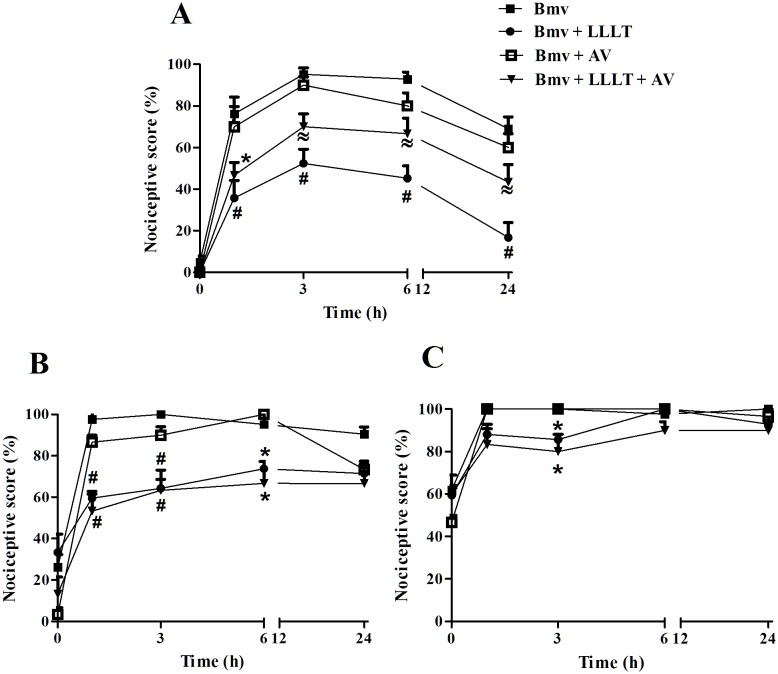
Effect of photobiomodulation on mechanical allodynia (A) or hyperalgesia (B and C) of mice. Animals were injected intraplantar with Bmv (1 μg; ■) and after 30 min and 3 h were treated with LLL at 685 nm (Bmv + LLLT; ●) or a combination of laser and antivenom (Bmv + LLLT + AV; ▼). Allodynia was measured by the mechanical response to tactile stimulation assessed by a von Frey filament of 0.407 g (3.61 filament; A). Hyperalgesia was measured by the mechanical response to nociceptive stimulation assessed by von Frey filaments of 0.692 g (3.84 filament; B) and 1.202 g (4.08 filament; C). Pain threshold was determined before (baseline values, time 0) and 1, 3, 6, 12 and 24 h after BmV injection. Animals injected only with Bmv or with a combination of Bmv+AV (□) were submitted to the same protocol. Each point represents the mean ± SEM of five to seven animals per group. (*p<0.05, ≈ p<0.01 or #p<0.001 vs Bmv group are indicated).

We initially investigated the effects of photobiomodulation on the allodynia and hyperalgesia induced by Bmv. We found that animals injected with Bmv showed significant mechanical allodynia and hyperalgesia when compared with baseline measurement taken before the test, as indicated by basal threshold in response to stimulation by von Frey filaments observed from 1^st^ h after Bmv injection up to 24 h (Fig 1). Photobiomodulation treatment applied 30 min and 3 h after Bmv injection reversed mechanical allodynia of mice in all evaluated times (Fig 1A). Regarding hyperalgesia, LLL was able to interfere with mechanical sensitivity evaluated by 3.84 filament in all evaluated times (Fig 1B) however, for the 4.08 filament the reversion of hyperalgesia was observed only at the 3^rd^ h of evaluation (Fig 1C). AV treatment itself did not interfere with mechanical sensitivity of mice (Fig 1).

### Effect of Photobiomodulation on Neuronal Activation

**Fig 2:**
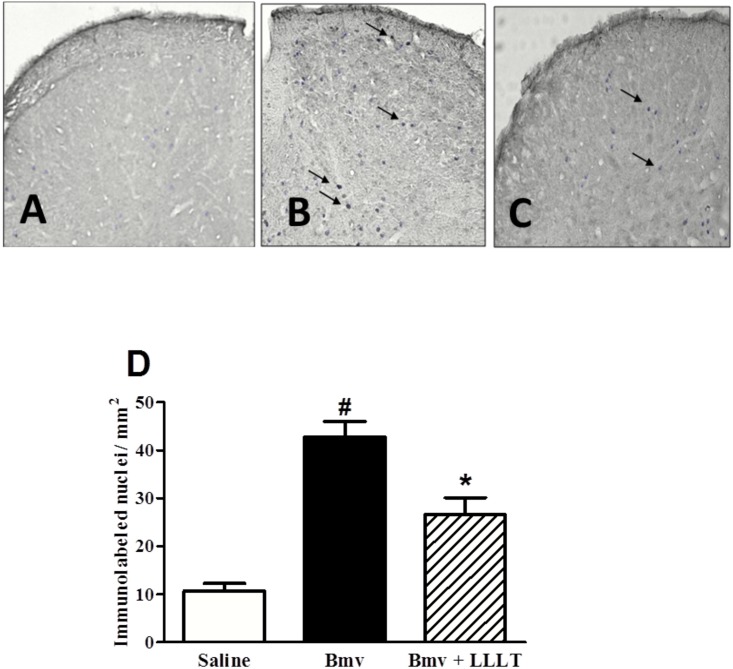
Fos immunolabel in the in the dorsal horn of the spinal cord (DHSC) of mice. Photomicrographs illustrating Fos immunostaining (arrows) in the dorsal horn of the spinal cord (DHSC) of mice injected intraplantar with saline (A), Bmv (B) or Bmv and laser at 685 nm (C). (D) Mean of the density of nuclei labeled for Fos protein in the DHSC of mice. Data shows the proto-oncogene immunoreactivity on the right side, ipsilateral to the paw irradiated. Values represent the mean ± SEM of 5 animals for group. Statistically significant differences vs. saline (^**#**^p<0.05) or vs. BmV (*p<0.05) are indicated.

As demonstrated in Fig 2, intraplantar administration of Bmv induced a significant increase of Fos immunoreactivity observed in the dorsal horn of the spinal cord of animals injected with Bmv (42.75 ± 3.26) when compared to the saline group (10.65 ± 1.61). Photobiomodulation treatment significantly decreased Fos expression (26.58 ± 3.58; Fig 2).

### Effect of Photobiomodulation on IL-6, IL-10 and TNF-α mRNA Expression

**Fig 3:**
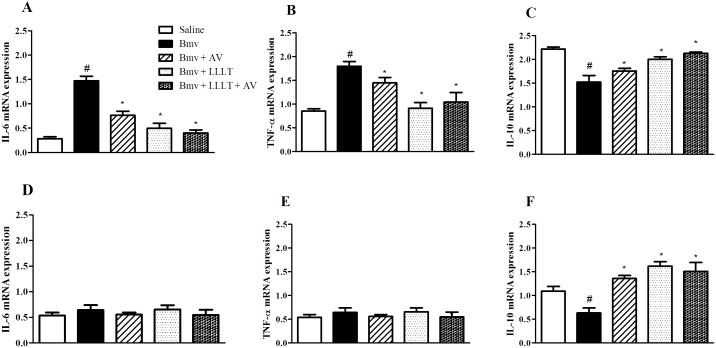
Effect of LLLT on IL-6 (A and D), TNF-α (B and E) or IL-10 (C and F) mRNA expression. mRNA levels of IL-6, TNF-α or IL-10 were evaluated by RT-PCR on samples from foot pad (A, B and C) or spinal cord (D, E and F) of mice injected intraplantar with saline or Bmv (1 μg) and treated or not with LLLT at 685 nm or antivenom (AV) or a combination of LLLT and AV were collected 6 h after treatments. Experiments were performed in triplicates. Data are expressed as means ± SEM of 5 animals from each group. Statistically significant differences vs. saline (^**#**^p<0.05) or vs. Bmv (*p<0.05) are indicated.

Cytokine production was evaluated on samples obtained from either spinal cord or footpad of animals previously evaluated at the nociceptive tests. As shown in Fig 3, the mRNA concentrations of IL-6 and TNF-α increased significantly at 6 h after Bmv injection in the footpad of mice when compared with control group (Fig 3A and 3B). After laser treatment, a significant reduction of both IL-6 and TNF-α mRNA levels was found. Moreover, treatment with AV did not significantly interfere with either IL-6 or TNF-α mRNA levels. However, concomitant treatment of mice with AV and phtobiomodulation decreased both IL-6 and TNF-α mRNA levels (Fig 3A and 3B). Furthermore, no changes on IL-6 and TNF-α were observed in samples from spinal cord of mice (Fig 3D and 3E). IL-10 mRNA levels were decreased after Bmv injection on both footpad and spinal cord of mice. Photobiomodulation treatment increased IL-10 levels in both footpad and spinal cord samples (Fig 3C and 3F). AV treatment did not interfere with IL-10 levels, however it prevented the decrease of this cytokine on samples from spinal cord (Fig 3F).

### Effect of Photobiomodulation on Kinin B1 and B2 Receptors mRNA Expression

**Fig 4:**
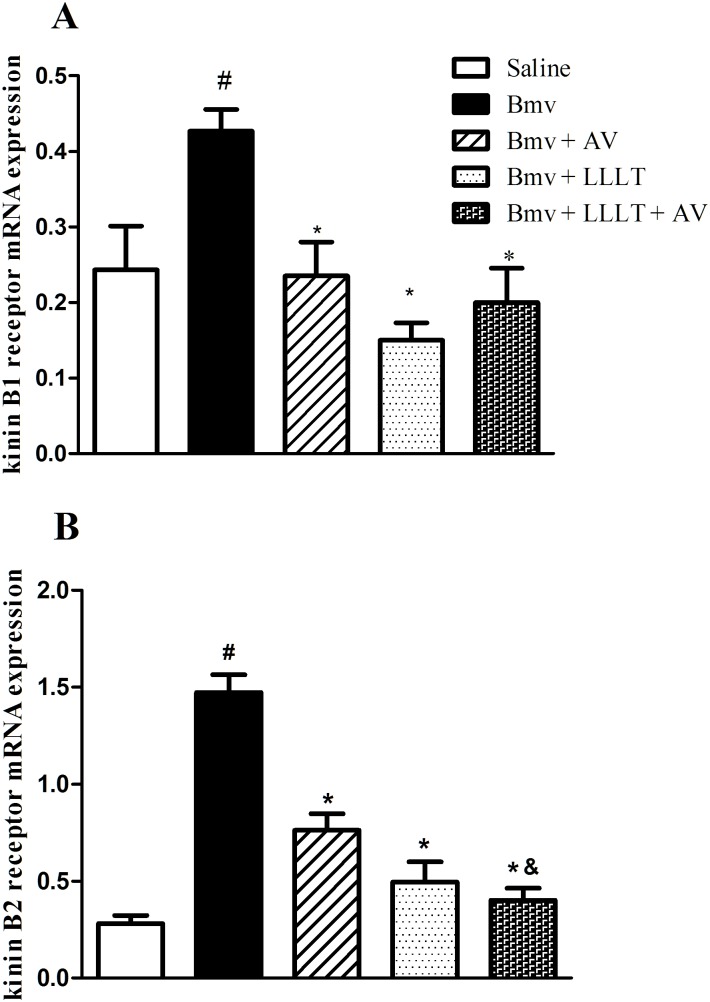
Effect of LLLT on kinin receptor mRNA expression. mRNA levels of B1 (A) or B2 (B) kinin receptors were evaluated by RT-PCR on footpad samples of the mice injected intraplantar with saline or Bmv (1 μg) and treated or not with LLLT 685 nm at or antivenom (AV) or a combination of LLLT and AV. Samples were collected 6 h after saline or Bmv injection. Experiments were performed in triplicates. Data are expressed as means ± SEM of 5 animals from each group. Statistically significant differences vs. saline (^**#**^p<0.05) or vs. Bmv (*p<0.05) or vs Bmv + AV (^&^p<0.05) are indicated.

A significant increase on mRNA expression of kinin B1 receptors was observed on Bmv-treated mice when compared to the control group (Fig 4A). LLLT, AV and the association of LLLT and AV induced a significant decrease of mRNA levels of kinin B1 receptors when compared with Bmv-treated animals (Fig 4A). Kinin B2 receptors mRNA expression was also significantly increased in envenomed mice paw when compared to control group (Fig 4B). Once again, LLLT or AV treatment decreased mRNA levels of B2 kinin receptors. More interestingly, the combination of LLLT and AV was more effective in decreasing B2 levels when compared with AV itself (Fig 4B).

## Discussion

The most effective treatment for venomous snakebites accidents is antivenom therapy. However, it is well known that such therapy is effective in neutralizing only the systemic effects of envenomation, without interfering with the severe local effects induced by these venoms [3]. Thus, given the importance framework triggered by local envenoming caused by bothropic venom and the incapacity of the antivenon to neutralize them, it is essential to investigate alternative therapies, with the greatest effectiveness in delaying progression and decreasing local symptoms of envenomed victims.

Among clinical symptoms induced by bothrops snakebites, local pain is a common and clinically relevant manifestation to the patient [28, 29]. Therefore, in this study, we investigated the capacity of photobiomodulation in reducing the nociceptive response caused by Bmv in mice footpad as well as the mechanisms involved. Herein, the intraplantar injection of Bmv induced mechanical allodynia and hyperalgesia. These results are in accordance with previous data demonstrating that Bmv induces potent mechanical allodynia and hyperalgesia in mice [24, 30]. Photobiomodulation applied 30 min and 3 h after Bmv reversed both mechanical allodynia and hyperalgesia. From these data, we confirmed that photobiomodulation, in fact, is effective in reducing Bmv-induced local pain. In our study, as in previous studies [24, 30], we observed that antinociception was not related to AV treatment, since it was not able to interfere with mechanical sensitivity of mice. Also, the association of LLLT and AV did not modify the effect of LLLT alone, reinforcing the therapeutic potential of LLL in treating local effects induced by bothrops venoms.

To better understand the capacity of photobiomodulation to decrease nociception, we evaluated the expression of Fos protein in the dorsal horn of the spinal cord of mice. The expression of proto-oncogenes from the c-fos, c-jun, and erg-1 family are extensively used as tools for the expression of enhanced activity of nociceptive neurons [20, 21]. Our results demonstrate that the intraplantar administration of Bmv induced a significant increase of Fos expression, observed in the dorsal horn of the spinal cord, which is characteristic of nociceptor activation. According to the results of this study, photobiomodulation not only significantly inhibited Bmv-induced mechanical allodynia and hyperalgesia, but also decreased nociceptor activation at the spinal level. More interestingly, we showed here that photobiomodulation is able to interfere with the transmission of Bmv-induced pain message to the central nervous system, reducing nociceptor activation at the central level. This result reveals sensory neurons as an important cellular target for photobiomodulation in the context of pain. In addition to nociceptor-mediated effects, other mechanism(s) may also take part in the antinociception observed in our experimental model. We hypothesized that photobiomodulation may reduce the inflammatory cytokines in the paw and spinal cord. Therefore, the next experiment was designed to further validate the proposed hypothesis.

It is commonly believed that proinflammatory cytokines such as TNF-α and IL-6 are involved in the pain process and that their peripheral and central levels are up-regulated in many pain models [31, 32]. In addition, as described in previous studies, Bothropic venom induces the accumulation of pro-inflammatory IL-6 and TNF-α cytokines in the local of venom injection, which contributes to the enhancement of local tissue damage [1, 33, 34]. Moreover, some studies suggest that the analgesic effect of LLLT may be due to the anti-inflammatory activity by the inhibition of inflammatory mediators [13, 35, 36]. Hence, to further analyze the mechanism by which photobiomodulation reduces nociception of mice induced by Bmv, the expression of pro-inflammatory IL-6 and TNF-α cytokines was evaluated on samples obtained from either footpad or spinal cord of animals. Our results showed that photobiomodulation was able to reduce IL-6 and TNF-α gene expression in the footpad of animals. Also, we showed that associated treatment of AV and LLLT induced the same decrease on IL-6 and TNF-α mRNA levels as the observed with LLLT alone. Moreover, no changes on IL-6 and TNF-α mRNA levels were observed in samples from spinal cord of mice, thus suggesting that inhibition of hyperalgesia depends on a peripheral inhibition of inflammatory cytokines. This result corroborates the study of Ferreira et al. (2005) [13] that proposed that the analgesic effect of LLLT involves the inhibition of hyperalgesic mediators.

Regarding IL-10, we observed that Bmv injection decreased IL-10 mRNA levels on both footpad and spinal cord samples. Also, LLLT increased IL-10 mRNA levels in both footpad and spinal cord. AV treatment did not interfere with IL-10 levels on samples from footpad of mice. However it prevented the decrease of this cytokine on samples from spinal cord. From these data, we confirmed that AV prevents systemic effects induced by Bmv however it did not protect against local hyperalgesia. IL-10 is considered a regulatory cytokine, related to the control of the inflammatory process due to its capacity of inhibiting the proinflammatory cytokine secretion [37]. Results presented herein suggest that laser irradiation was able to modulate the expression of this regulatory cytokine, both in the local of venom injection and in the spinal cord, and it appears likely that this modulation plays a role in the anti-nociception observed after bothropic venom in response to photobiomodulation.

To further analyze the mechanism by which photobiomodulation reduced Bmv-induced nociception, we evaluated the kinin receptors levels in the footpad of mice. Both kinin B1 and B2 receptors, evaluated here, play a central role in the pathophysiology of inflammation [38]. Kinin B2 receptors are broadly and constitutively expressed in most tissues, whereas B1 receptor is weakly expressed in most tissues under basal conditions but strongly upregulated following inflammation [39]. The involvement of bradykinin on Bmv-induced hyperalgesia and edema has been demonstrated [7, 40]. In addition, it was already demonstrated that the kinin B2 receptors are involved in hyperalgesic response induce by *B*. *jararaca* and *B*. *asper* venoms [22, 41]. Our results demonstrate that both B1 and B2 kinin receptors are increased in the footpad of animals injected with Bmv. Among the treatments, we found that both LLLT and AV were able to reduce the expression of B1 and B2 kinin mRNA levels. However, the association of LLLT and AV showed greater effectiveness in reducing B2 kinin receptors. Considering that kinin receptors are important mediators on *Bothrops*-induced hyperalgesia [22, 23] it is feasible to suggest that photobiostimulation reverses Bmv-induced hyperagesia, at least in part, by modulating bradikinin receptors involved in the process.

We conclude that photobiomodulation with low level laser is effective in decreasing nociceptor activation at the spinal level. Moreover LLL is effective in modulating pro- and anti-inflammatory cytokines as well as kinin receptors at mRNA transcriptional level. These effects, at least in part, contribute to the decrease of hyperalgesia observed after Bmv. Photobioestimulation with the parameters used herein should be considered as a potential therapeutic approach for the treatment of local effects of Bothrops snakebite.
